# A deep learning based multimodal interaction system for bed ridden and immobile hospital admitted patients: design, development and evaluation

**DOI:** 10.1186/s12913-022-08095-y

**Published:** 2022-06-21

**Authors:** Muhammad Nazrul Islam, Md Shadman Aadeeb, Md. Mahadi Hassan Munna, Md. Raqibur Rahman

**Affiliations:** Department of Computer Science and Engineering, Military Institute of Science and Technology, Dhaka-1216, Bangladesh

**Keywords:** Hospital cabin, Multimodal interactions, Deep learning, Computer vision

## Abstract

**Background:**

Hospital cabins are a part and parcel of the healthcare system. Most patients admitted in hospital cabins reside in bedridden and immobile conditions. Though different kinds of systems exist to aid such patients, most of them focus on specific tasks like calling for emergencies, monitoring patient health, etc. while the patients’ limitations are ignored. Though some patient interaction systems have been developed, only singular options like touch, hand gesture or voice based interaction were provided which may not be usable for bedridden and immobile patients.

**Methods:**

At first, we reviewed the existing literature to explore the prevailing healthcare and interaction systems developed for bedridden and immobile patients. Then, a requirements elicitation study was conducted through semi-structured interviews. Afterwards, design goals were established to address the requirements. Based on these goals and by using computer vision and deep learning technologies, a hospital cabin control system having multimodal interactions facility was designed and developed for hospital admitted, bedridden and immobile patients. Finally, the system was evaluated through an experiment replicated with 12 hospital admitted patients to measure its effectiveness, usability and efficiency.

**Results:**

As outcomes, firstly, a set of user-requirements were identified for hospital admitted patients and healthcare practitioners. Secondly, a hospital cabin control system was designed and developed that supports multimodal interactions for bedridden and immobile hospital admitted patients which includes (a) Hand gesture based interaction for moving a cursor with hand and showing hand gesture for clicking, (b) Nose teeth based interaction where nose is used for moving a cursor and teeth is used for clicking and (c) Voice based interaction for executing tasks using specific voice commands. Finally, the evaluation results showed that the system is efficient, effective and usable to the focused users with 100% success rate, reasonable number of attempts and task completion time.

**Conclusion:**

In the resultant system, Deep Learning has been incorporated to facilitate multimodal interaction for enhancing accessibility. Thus, the developed system along with its evaluation results and the identified requirements provides a promising solution for the prevailing crisis in the healthcare sector.

**Trial Registration:**

Not Applicable.

## Background

Caring for patients admitted in hospitals is a difficult and challenging task. Patients are generally admitted to a hospital cabin when they have serious health problems which can often be life threatening. They may also be admitted for less serious disorders that require constant monitoring and proper rest. Also several treatment practices can not be applied to a patient unless he/she is admitted in a hospital cabin for some definite interval of time. Most of these patients reside in bedridden and immobile conditions when admitted to hospital cabins. Furthermore about 40% of these admitted patients belong to the elderly population with the average age being above 65 years [1]. Also there are people with disabilities who are admitted in hospitals; and research has shown that they have an increased length of stay [2]. The movements and interactions of hospital admitted patients with their surroundings become restricted due to serious health conditions, age, disability and prescribed medical instructions. Hence, such patients require external support almost 24 hours a day for performing even the minimal day to day tasks and thus become highly dependent on the healthcare professionals for support.

Recent studies found that the healthcare providing sector is severely understaffed and the health care professionals work under extensive workload throughout the world [3, 4]. This further deteriorates the quality of healthcare for the patients [5]. Research also shows that pressure on the healthcare sector is prevalent in both developed countries [6] as well as in the developing countries [3]. Again, due to the covid-19 pandemic, the situation has further deteriorated [7, 8]. However, developed countries like Finland, Canada, England, Ireland, New Zealand and Japan have focused on designing patient care solutions in order to improve the quality of healthcare [6]. But though researchers have focused on developing systems to aid hospital admitted, bedridden and immobile patients with limited interaction capacity (for example, patient monitoring systems, brainwave based interaction systems, hand gesture based interaction systems, etc.) [9–11], very few work has been conducted to identify the requirements of such patients and develop a digital solution to address their requirements.

Again, in order to mitigate this healthcare crisis, the requirements of a patient in hospital cabins are to be clearly identified and understood. Based on the identified requirements, a potential solution can be designed and developed to aid the admitted patients along with the healthcare practitioners.

Recent studies show that, deep learning based systems have been gaining popularity in the healthcare sector due to their efficiency, accuracy and scalability when considering large sized data sets [12, 13]. Several systems deployed in the medical sector have been developed using deep learning technologies (for example, medical image analysis, disease prediction, etc.) [14, 15]. But not much work has been done using deep learning considering immobile and bedridden patients admitted in hospital cabins.

Hence, the objectives of this research are: firstly, to understand the stakeholders’ (patients, nurses, doctors) requirements (mode of interaction, system features, functionalities, etc.) for developing a hospital cabin control system for the bedridden and immobile patients; secondly, to design and develop the hospital cabin control system as per the identified requirements adopting deep learning technologies and finally, to evaluate the developed system through empirical study.

Thus, the contributions of this paper are as follows.Firstly, this research explicitly focuses on identifying the requirements of hospital admitted patients and concerned healthcare practitioners. The data collected during the requirements elicitation process will be of help in designing systems for hospital admitted patients. Secondly, the algorithms used for this system are based on deep learning and hence the efficiency and accuracy of the system in real time has been enhanced. Finally the proposed system supports multiple modes of interaction and hence people with different sicknesses, disabilities and complications can be benefited by the system.

A search has been conducted in the major scholarly databases including Google Scholar, IEEE Xplore, Springer Link, ACM digital library and ScienceDirect. While searching, keywords like “Hospital Patient”, “IoT Healthcare”, “Hospital Cabin”, “Patient Care System”, etc. were used. The year range used while conducting the search was 2016 to 2021, however, few articles out of this time range have been selected as well due to having correlation with our research.

A number of digital systems have been developed for providing healthcare services for the immobile, sick, disabled and elderly people [10, 11, 16–20]. Kanase and Gaikwad [21] proposed a cloud based system where different sensors like light, temperature, sound and saline level sensors were used to collect data from the patient’s room. An app and a website were developed for displaying the sensor readings and for controlling the patient’s room appliances by the concerned doctors, nurses and staff. This research demonstrated how different electronic appliances can be operated in a hospital cabin by individuals from a distant location. The study further presented how different necessary data can be transferred from the patient’s end to the caregiver’s end. But in this proposed system no control was placed in the hands of the patient. Sari and Maher [16] proposed an architecture of a smart healthcare system that collected data from patients through different activity sensors and medical sensors and sent the data to a central database for monitoring. Whenever an anomaly was detected in the data collected by the sensors, an emergency alert would be sent to the concerned healthcare practitioners. Their research highlighted the necessity and importance of an emergency and real time alarm generation system using which swift medical response can be ensured for the patients. Similarly, Kamruzzaman [22] and Saha et al. [9] proposed system architectures for patient monitoring and emergency alarm generation. Their proposed architectires further emphasized the significance of transfer of data between the patients and the caregivers in real time for emergency as well as normal medical response.

A number of studies were also carried out to develop care-giving systems using the brain waves of the patient. For example, Gao et al. [10] developed a Brain Computer Interface based electrical apparatus controller. The developed system showed how using alternate means of interaction can benefit disabled patients to be able to control their surroundings including television, air conditioner, etc. Wolpaw et al. [23] developed a BCI based system for communication and control for people with disabilities. Their research further portrayed how alternate means of interaction can benefit disabled, elderly and sick patients in performing both communication and control related functions. Aadeeb et al. [24] designed a BCI and Deep Learning based hospital cabin management system for helping sick, disabled and bedridden patients in controlling their surroundings. The system design consisted of a Graphical User Interface (GUI) using which the patient can control the surrounding electrical apparatus. Their article not only highlighted the significance of alternate means of interaction for disabled people, but also showed how a GUI can further enhance the alternate means of interaction. Moreover, the design proposed in their system also made use of Deep Learning technologies and showed how Deep Learning can be used to make alternate means of interaction more accurate, efficient and effective. Chun-Lo et al. [25] implemented a BCI based system where the patients brain waves were being used detect the direction of the patient’s eye gaze. In their work, an instance of combined interaction has been showed where eye gaze based interaction and Brain computer interaction have been used together. In their developed system, a graphical user interface (GUI) is positioned in front of the user where different GUI elements blink with different frequencies. The different frequencies in the GUI helps to develop different brainwave signals in the patient’s brain which can be used to detect in which direction the user is staring at. Based on the detection, the surrounding environment around the patient can be controlled by the system’s control program. Along with presenting a combined interaction approach, this research further emphasized on the necessity of a GUI in developing interaction systems for the disabled people.

A number of studies were conducted to help disabled or sick patients by detecting and tracking different organs. These studies demonstrate how kinesics and para language [26] can be utilized for developing healthcare systems for the disabled people.

A number kinesics based researches utilized the use of hand gestures for developing systems based on alternate means of interaction. Jagdish et al. [11] developed a system for disabled users to call for assistance that includes nine options (calling doctor, calling family, calling for fruits, calling nurse, calling for emergency, calling for food, calling for bathroom, calling for water and calling for medicine). Disabled users can interact with the system using hand movement. Using hand tracking a cursor is moved and using hand gesture recognition an event is triggered in the system. Their research is one of the earliest works where computer vision was used for ensuring hand gesture based interaction for hospital admitted patients. Their work also showed how computer vision based interactions eliminate the necessity of hardware sensors. However in this system, the user was not provided the option to control the surrounding environment around him/her (for example, turning on a fan or light). Kakkoth and Gharge [27] designed and developed a system where hand gestures are detected using a camera in real time and transformed into texts and sounds. Their research showed how a hand gesture based interactions can be utilized as a means of communication for patients. Chattoraj et al. [28] developed a hand gesture based real time image processing system where hand gestures were detected and classified for sign language interpretation. In this study also, hand gesture based interaction helped ensure alternate means of communication for disabled people. Tam et al. [29] developed a hand gesture based controller. The controller consisted of a Electromyographical sensors which helped in detecting muscle movements. The Electromyographical sensors were used to generate features for a convolutional neural network. The convolutional neural network helps to classify the hand gestures with 98.15% accuracy. This system further showed how deep learning techniques can significantly improve hand gesture based interactions. However, unlike the previously mentioned hand gesture based interactions, this research did not use computer vision and hence required to incorporate the use of sensors.

Again, many researches were conducted where the patients’ eyes and head movements were utilized using computer vision for healthcare purposes. Hutchinson et al. [30] developed an eye gaze based system named Erica for the sick and disabled people. The system tracks the user’s eyes and predicts where the user is starting at on the monitor. Their research demonstrated how alternate means of interaction can be achieved by detecting and tracking very small organs of the human body like eyes and how a GUI can help to improve the interaction. Similarly, Sunny et al. [31] developed a wheelchair mounted robotic arm that can be controlled using eye gaze. In this system also, a GUI was used and it was placed on the wheelchair along with the robotic arm. By gazing at different graphical elements of the GUI, the robotic arm can be controlled. This system further successfully demonstrated how alternate means of interaction using just eyes can be further improved with the help of graphical user interfaces. Kim and Ryu [32] proposed a system for the hands-free control of a computer. Here, PC camera was used to track head movements, then these movements were translated into cursor movements onto a computer screen. The proposed system is an alternative for people with spinal cord injuries and other special needs. This research displayed how simple bodily motion can be interpreted as robust controlling means for the disabled people.

Several of the alternate means of interactions were developed based on the interpretation of patient’s facial features and computer vision. Khan et al. [33] developed and evaluated a nose tracking cursor control system for the disabled patients unable to use their hands. A user can use this system by moving a cursor with his/her nose and by showing his/her teeth to execute a cursor click. The developed system was evaluated at a hospital environment where disabled people unable to use their hands took part in testing the system. This system not only showed how nose and teeth movement can be interpreted for alternate means of interaction but also demonstrated the importance of system evaluation for the justification of such systems. Islam et al. [34] developed and evaluated another nose tracking cursor control system but it was developed for using Android smartphones. A disabled user unable to use his/her hands can use the system to perform all the basic touch operations and button operations. Their work showed how alternate means of interaction can implemented in different platforms to benefit the disabled people and enhance their accessibility.

Based on the literature review, it has been observed that a number of researches have been carried out to aid the healthcare sector. The proposed systems were successful to fulfill their own objectives. But very few studies have been conducted that explicitly focused on understanding the conditions, challenges and needs of bedridden and immobile patients admitted in hospital cabins. And though a number of researchers proposed care giving systems, very few of the systems were proposed in the context of patients admitted in hospitals. Also, most of the interaction systems developed in previous researches focused on incorporating single mode of interaction without considering the patients at different levels of the disability spectrum. Furthermore, very few patient care systems were found to be developed adopting deep learning technologies. Thus, this research focuses on understanding the requirements and challenges faced by both hospital admitted, bedridden and immobile patients as well as healthcare practitioners; designing a system solution to address the requirements; implementing the system solution and finally testing and evaluating the implemented system.

## Methodology

To attain the research objectives, this research was conducted in three phases. In the first phase, the requirements elicitation study was done to reveal the requirements of the hospital admitted, bedridden and immobile patients as well as of the healthcare practitioners. In the second phase, based on the identified requirements, a multimodal interaction system was designed and developed. In the final phase, the system was evaluated to test the usability, efficiency and effectiveness of the system. In this section the procedures followed in the three phases have been described. The outcomes of the corresponding procedures have been presented in the “Results” section.

### Requirements elicitation study

The requirement elicitation study was conducted following a semi-structured interview approach from April to May 2021 in Dhaka, Bangladesh. The purpose of the study was to identify the potential problems faced by healthcare practitioners and by the patients. In the following subsections, the information about the participants who took part in the study is discussed, after that, the description of the study procedure is provided. The findings from the study are presented in the “Results” section.

#### Participants’ profile

A total of 18 participants were involved in the requirement elicitation process. For recruiting the participants we at first contacted the 2 intern doctors and 5 ex-patients who were available in our social network. Then following the snowball sampling method [35], we came in contact with the remaining participants. 10 of the 18 participants were ex-patients who had been admitted in hospitals before for a duration of 1-6 weeks. The ex-patients’ profiles are briefly presented in Table 1. The remaining participants included two doctors, three intern doctors and three nurses. One of the doctors was a gynaecologist with 9 years of experience. The other doctor was a cardiologist with 5 years of experience. The three intern doctors included in the study were recent graduates from different medical colleges and newly deployed into the healthcare sector. The three nurses were nursing school graduates having average experience of 5±1.7 years.

**Table 1:** Ex-patients’ profile

SL	Age	Gender	Time Spent in Hospital Cabin	Last Reported Sickness
1	45	Male	14 days	Covid-19
2	56	Male	2 days	Cataract in eyes
3	69	Female	45 days	Uterus Infection
4	47	Male	7 days	Heart Attack
5	56	Female	7 days	Dengue
6	37	Female	2 days	Urine Infection
7	15	Female	4 days	Dengue
8	67	Male	35 days	Heart Attack
9	23	Male	14 days	Covid-19
10	16	Male	7 days	Motorbike Accident

#### Study procedure

The study procedure was conducted using a semi-structured interview. An “informed consent” form was signed by each of the participants to participate in the interview sessions to ensure that the participation was voluntary and that their identity will not be disclosed and the data will be used solely for research purposes. The interview was conducted in Bengali. The interview questions were different for healthcare practitioners (doctors, intern doctors and nurses) and ex-patients.

In the case of the ex-patients,at first we asked them about their demographic information such as age, illness, last time they were admitted in a hospital,etc. After that, we asked them to describe the room in the hospital in which they were admitted. We followed up by asking about the management of the hospital room in which they were admitted. Next we asked them about the number of times the healthcare practitioners came to them for follow ups or check ups in a day. Then we requested them to tell us whether they were satisfied with the existing systems in the hospital for admitted patients.After which, we asked them about the problems faced by them while they were staying admitted in the hospitals. Then we inquired from them whether they would like to use a digital system solution to solve their problems. We queried about the types of functionality they would desire from a digital system solution. We also inquired about how they would like to interact with a digital system solution. During the interview, we tried to ensure that the ex-patients were comfortable and encouraged to answer our questions. At the end of the interview, we asked the ex-patients if they wanted to add anything we might have missed.

In the case of the healthcare practitioners (i.e. the doctors,interns and nurses) we at first asked them to describe the condition of the patients admitted in hospitals from their point of view. We then asked them whether they face any problems while dealing with the admitted patients. Then we inquired from them whether they were satisfied with their daily workload and the working hours. Then we requested them to tell us about the challenges they face while dealing with patients. After that we asked them for their views about a digital system solution for dealing with the admitted patients. We also queried them to understand whether such a digital system solution would be feasible or not. The audio recording of the interview was done for each of the participants for future reference with their kind consent. Each of the interviews lasted for about 15 to 20 minutes.

Though the interviews were conducted using the Bengali language, one of our authors transcribed the interview into English. Then, the interview data was analyzed using the Six Phase Approach for Thematic Analysis [36, 37]. The outcome and findings from the thematic analysis is presented in the next section.

### Design and development of the system solution

In order to address the requirements obtained as the result of the requirements elicitation process, a system solution was designed and developed to aid the stakeholders associated with hospital cabins and wards. The proposed system required to be capable of detecting and tracking human face, human hand and human nose. Furthermore, the system also required to be able to classify whether a patient is showing teeth or not and also to classify two distinct hand gestures showed by a patient.

For the purpose of detection and tracking and for classification, convolutional neural networks (CNN) [38] have been used in this research. Two different types of CNNs have been adopted in the proposed system. The first type of CNN has been used for classification purposes and is referred to as classification CNN. The second type of CNN has been used for the purpose of detection and tracking using the YOLO algorithm [39] and this type of CNN is referred to as YOLO based CNN. In this section, the procedures based on which the proposed CNNs work has been described along with the underlying mathematical principles. In the “Results” section, the resultant CNNs developed and applied in the system have been discussed.

In order to train [40] the CNN models, at first dataset preparation has been done. This involved several steps, including image data collection, image data labelling, image resizing, image cropping, image scaling, reducing image color channels, etc. The image data was further divided into two subsets for training and validating the CNN models. Moreover, the images were randomized and organized into batches for training the CNNs. The resultant image dataset obtained as a result of the mentioned steps have been described further in the “Results” section.

The CNN for classification consist of one Input layer [41], multiple convolutional layers [42] and multiple pooling layers [43]. Furthermore, the classification CNN also consist of a Dense layer [44] which is not used in the YOLO based CNN.

The Input layer is used for providing an image as input to the CNN. This layer is used to pass the image onto the subsequent CNN layers as an input feature map [45]. The Convolutional layer takes in a 3 dimensional feature map as input and performs the convolution operation [46] on the feature map following Eq. 1, where *F* denotes a feature map, *I* represents a convolution kernel, *x* and *y* represents the co-ordinates of the region of the feature map on which the center of the kernel is located and *i* and *j* denotes the co-ordinates around the center of the kernel.

1 $$F_{o}I(x,y) = \sum_{j=-N}^{n}\sum_{j=-N}^{n} F(i,j) I (x+i,y+j)$$

The pooling layer is used to reduce the width of a feature map. It consists of a single kernel which operates on the input feature map. The new width of the feature map can be calculated using the Eq. 2. Furthermore, due to its size reduction capabilities, the pooling layer is also used to convert 3 dimensional feature maps to 1 dimensional feature maps.

2 $$new Width = \lfloor \frac{width Of Feature Map - kernel Size}{strides} + 1 \rfloor$$

The Dense layer takes one dimensional feature map *x* as an input parameter and multiplies it by its weight values *w*. The dense layer also consists of a bias value *b* which is added to the product obtained by the weight multiplication. The dense layer performs the weight multiplication and the bias addition with the input features using the Eq. 3.

3 $$y=wx^{T}+b$$

Each layer of a CNN consists of an activation function [47] which is used to alter the output from a CNN layer. In the proposed CNNs, relu activation function is used in the convolutional layers for processing the feature maps given as outputs from these layers and softmax activation function has been used in the final Dense layer for classification purposes. The relu activation function and the softmax activation functions follow Eqs. 4 and 5 respectively.

4 $$y=max(x,0)$$

5 $$\sigma(x)_{i}=\frac{e^{x_{i}}}{\sum_{j=1}^{k}e^{x_{i}}}$$

The architecture of the CNNs used in the proposed system for classification has been elaborately described in the next section.

The CNNs proposed for running the YOLO algorithm contains convolutional layers as well as pooling layers. The difference between the classfication CNNs discussed above and the YOLO algorithm based CNNs is that, instead of using a Dense layer as the final layer, a convolutional layer is used in the YOLO based CNNs. A 3D feature map is given as output by this convolutional layer. The 3D feature map consists of several rectangular grids and each grid corresponds to a definite region of the input image. This 3D feature map is the input argument for the YOLO algorithm. The final 3D feature map grids can be represente by a vector*z*_i_. The value of each vector *z*_i_ follows Eq. 6.

6 $$z_{i}=[p_{i},w_{i},h_{i},x_{i},y_{i}]$$

In the Eq. 6, *p*_i_ is the probability that an object has been detected in the region represented by the vector *y*_i_. The detected object can be bounded by a rectangular region which starts at the point (*x*_i_,*y*_i_) and has height and width of *h*_i_ and *w*_i_ respectively.

Furthermore, non max suppression algorithm [48] has been used on the vectors so that no two vectors of the final 3D feature map can claim to have the same object. The non max suppresion algorithm uses the metric Intersection over Union *J(A,B)* which follows the Eq. 7.

7 $$J(A,B)=\frac{|A \cap B|}{|A \cup B|}$$

### System evaluation procedure

The main purpose of evaluating the developed system was to test the usability, efficiency and effectiveness of the system. In the “Results” section, the findings from the evaluation study has been presented and described. For this, the prototype of the system was tested by real patients admitted in hospitals.

The initial communication with these patients were possible due to the kind help from the doctors who took part in the requirement elicitation process discussed in section 3. Before performing the testing of the system, permission was taken from the patients as well as their guardians. A consent form was signed by the guardians of the patients as well. Furthermore, the patients along with their guardians were ensured that the information about them will be used solely for research purposes and they could withdraw from the testing at any time if they wanted to.

#### Participant’s profile

A total of 12 participants aged between 15 to 70 years with a mean of age of 47 and std-dev of 15.64 took part in the testing process. 6 of the participants were male and the other 6 were female. These people had prior experience with using mobile or desktop applications. All the participants were admitted to the hospital for various health issues. Table 2 shows the information about the participants.

**Table 2:** Participant’s profile

Participants	Gender	Age	Reasons to admit in hospital	Prefered mode of Interaction
P1	Male	45	Heart Attack	Voice based interaction
P2		56	Cataract in eyes	Hand based interaction
P3		69	Kidney Failure	Hand based interaction
P4		47	Dengue	Nose-teeth based interaction
P5		15	Dengue	Voice based interaction
P6		37	Uterus Infection	Nose-teeth based interaction
P7	Female	44	Broken Hand	Nose-teeth based interaction
P8		70	Stroke	Voice based interaction
P9		49	High blood pressure	Voice based interaction
P10		57	Heart Attack	Hand based interaction
P11		52	Broken leg	Nose-teeth based interaction
P12		23	Malaria	Nose-teeth based interaction

#### Study procedure

The experiment was conducted with some specific equipment like a camera, smartphone with phone holder and a laptop. The laptop was used to display the user-interface so that users can interact with it. The laptop was also used for collecting participant’s voice data for voice based interaction and image data for hand based interaction techniques. The camera was used to get image data of face and nose to make interaction for nose-teeth based interaction techniques. For each participant, an “informed consent” to participate in the study was taken. Each of the patients were at first provided with a small brief and demonstration of the systems. Then they were requested to choose their preferred mode of interaction.

The tasks that were performed by the patients were divided into three main categories. The first category of tasks were *call* related tasks, which included the tasks of calling a family member and calling a nurse. The second category of tasks was related to surrounding control. It included 6 tasks which are turning light on/off, raising/lowering temperature and turning on/off fan. The third category of tasks was related to controlling the bed. For this reason, the prototype bed that was described in the subsection “The User Interface and Prototype Bed” was used as it was not possible for us at that moment to build a custom motor controlled hospital bed. Table 3 further describes these three task categories.

**Table 3:** Task summary

Task No	Task Scenario	Operations that were needed to be done
T1	An elderly patient is admitted into a hospital cabin and he can’t even walk properly. He needs assistance to visit the toilet and sometimes he feels terrible with the room’s environment. Again, sometimes he misses his family so much and he has to wait for the nurse each time to get help or to call his family.	Press or Say “call family” Press or Say “call nurse”
T2	A patient is admitted into the hospital cabin and he has to rest in the hospital bed. Sometimes it is hard for him when he can’t control the room’s environment like - turning on light or fan, changing temperature, etc. He has to wait for the nurse and ask her to do these simple things for him.	Press or Say “Turn on Light” Press or Say “Turn off Light” Press or Say “Increase temperature” Press or Say “Decrease temperature” Press or Say “Turn on Fan” Press or Say “Turn off Fan”
T3	A patient whose both hands are severely injured is admitted in the hospital cabin. He can’t use his hands at all and is fully dependent on the nurse to control his bed conditions like - raising and lowering bed’s head or leg, raising and lowering left or right side of the bed, etc.	Press or Say “Raise Bed Head” Press or Say “Raise Bed Leg” Press or Say “Raise Bed Left” Press or Say “Raise Bed Right” Press or Say “Lower Bed Head” Press or Say “Lower Bed Leg” Press or Say “Lower Bed Left” Press or Say “Lower Bed Right”

The experiments were conducted separately in different sessions according to the patients’ convenience. For the purpose of quantitative analysis and determining effectiveness, efficiency and usability of the system, necessary data including number of attempts, task completion time and successful execution rate were collected and stored. When the experiment was completed, a set of questionnaires were asked about their overall experience on this system. Their opinions and feedback were compiled later on for qualitative analysis.

## Results

### Outcomes of the requirements elicitation study

The findings from the study procedure are reported by first describing the physical problems faced by the ex-patients. After that the mental distress faced by the ex-patients due to their detachment from family are reported. This is followed up by describing the situation of the ex-patients in various emergency situations. After this, the workload and pressure on the healthcare practitioners is discussed. Lastly the expectations of the participants about the digital system solution is described.

#### Physical problems faced by the patients

During the interview, while probing the problems faced by the ex-patients during their stay in hospital, we have identified the following physical problems faced by them.

##### a) Problems faced due to lying in bed:

Patients admitted in hospital have to spend long hours lying down in the bed. This causes the muscles to become sore. The patients who are unable to move, for example patients who became injured due to an accident, are forced to stay in bed in the same position throughout the day. One such ex-patient said: “Since both my legs were plastered and placed by the doctor in a suspended position, I was unable to turn sideways while lying on the bed. This resulted in causing pain in my neck and back. I was also feeling uncomfortable in my hip muscles and behind my head. The bed I was placed in was not a machine controlled bed and had to be adjusted by the nurses manually. Adjusting the bed by raising the sides or by raising the bed near my shoulders could alleviate my sufferings to some extent. Several times throughout the day, I fell into situations where I was facing severe discomfort and no nurse was available at that moment to adjust the bed for me.” In modern hospitals, the hospital beds can be controlled with the help of a remote controller. Using the controller, different sides of the bed can be raised or lowered. The controller can be used by the patients or by the nurses. But such controllers require the use of hands. Patients whose hands have been amputated or patients who are unable to use their hands cannot make use of this remote controller. Though among the participants there were no ex-patients with problems in their hands, this problem was confirmed by the healthcare practitioners. Regarding this matter, one intern doctor said: “There are several patients who are unable to use their hands. For example amputees or patients with plastered arms. These people face difficulties in doing the simplest tasks without external assistance. Regarding your concern about bed control, I do agree with you. The bed controller is indeed a meaningless device for these types of patients.” During the requirements elicitation process, we also found that, due to intense workloads, it is not always possible for the nurses to adjust the bed for the patients. At times, they become busy with higher priority tasks and hence they become unable to help patients with issues like bed control. Regarding this a nurse had the following opinion: “At times I alone have to be in charge of a ward with 20 patients. During those times the workload becomes intense. And sadly I become unable to attend to the needs of everyone at the same time.”

##### b) Problems with light and temperature:

Most of the ex-patients who took part in our study mentioned their discomfort due to the temperature of the room. Since a common symptom for almost all the patients was fever, they frequently felt the necessity to adjust the temperature of the room. One ex-patient who was admitted in a cabin with air conditioning facilities said the following regarding this matter: “The thermostat switch was embedded in the wall. Since I was in a very weak condition, it was not possible for me to get out of bed and change it myself. Every time I had to seek the assistance from a nurse or a doctor regarding this matter.” Even in rooms without an air conditioning system, where no thermostats are present and electrical fans are used, the patients face problems. Regarding this one ex-patient opined that: “The fan’s switch was very far away from my bed. Since my body temperature was frequently changing throughout the day. Many times I needed to adjust the speed of the fan. And it was tiresome to get up every time to do this” Similar problems were faced by the ex-patients due to their difficulty in controlling the lights in the room. Regarding this one ex-patient said the following: “At night, the lights in my room caused me trouble while I was trying to sleep. I used to feel very weak and uncomfortable and tried to sleep as soon as I had taken my medications at night. Many times the nurses around me used to forget to turn off the lights as it was still very early. I really wished that the light switch was near to my bed”

#### Mental distress due to detachment from family

During the interviews, it was identified that many of the ex-patients felt homesick during their stay at the hospital. Spending time alone in hospital cabins for several hours every day resulted in mental distress due to feelings of loneliness. Regarding this, one elderly ex-patient shared her experience: “I am a very jolly minded person and I really love to talk and communicate with people. But during my stay in the hospital, I had to spend several hours alone. The doctors and nurses used to come during their scheduled times, but during the other times I was all by myself. I also face difficulty in using the mobile phone I have. Though I am able to answer a call, I find it difficult to make a call due to my old age and sickness.” The ex-patients also faced other types of mental distresses including fear, anxiety and sadness. All the ex-patients believed that their detachment from family members further deteriorated their state of mind. Though the ex-patients who were able to use mobile phones could communicate easily with their near and dear ones, the ones who are unable to use mobile phones would not have the same benefits. Regarding this one of the doctors said the following: “I have treated several patients who are unable to use mobile phones. Such patients include amputees, accident victims, people with neurological disorders,etc. As they are unable to make or receive phone calls, they become bound to spend lonely times with anxiousness, fear and sadness. Though we, the health care practitioners try our best to keep them mentally stable and happy, we are not able to mentally encourage them all the time due to our extensive workload.”

#### Problems faced during emergency situations

During the interview, few of the ex-patients were identified who had problems going to the washroom and had asthma issues. These problems can create emergency situations for the patients at times. One elderly ex-patient explained his experience as: “I used to call a nurse to go to the washroom but it was very difficult for me to wait till the nurse came. It was an emergency situation for me because nurses were not always available as they had to look after a lot of patients. Again, at this old age, it is really hard to maintain my bathroom schedules”. Another ex covid-19 patient explained his problem while being admitted in hospital as: “I had respiratory troubles and a nurse was assigned to look after me. But as there was a shortage of nurses, she had to look after some other patients too. Sometimes I had an emergency situation where I had difficulty breathing and needed help immediately. The nurse tried to help as soon as possible but sometimes it took time and I had to help myself in those critical situations. It would be better if I could get immediate support during those emergency periods”. The emergency related issues were further discussed with the doctors and nurses. One doctor said: “We have always tried to help our patients when they need us but sometimes it gets really difficult to determine the emergency situation of the patients. Emergency periods may come for multiple patients who are under the surveillance of a single nurse. At that time, if the nurse can determine an emergency situation for more than one patient, she calls for immediate help. After that, more nurses and doctors come to help”. The explanation of this situation from the nurse was quite similar. The problem was communication issues between patient and nurse as checking multiple patients’ condition is very hard for a single nurse especially when they had a critical situation.

#### Workload on the healthcare practitioners

During the interview with doctors, nurses and intern doctors, their workload was exposed. Firstly one doctor was interviewed. When he was asked about his workload, he said: “I have to maintain a very busy working schedule daily starting at 8:00 am and it may end at 7:00 pm without any significant break. This schedule may vary depending on the situation. Sometimes I have to do overtime if there are any emergency cases”. The intense nature of working schedules was also accepted by several other doctors as well and they agreed that they have to invest a huge amount of time at the healthcare center. One of the intern doctors agreed to explain his daily workload. He said: “We have 2 types of working hours - Day shift and Night shift. Both are at least 15-hours long and full of workloads. Again, we have to take government recruitment exam preparation whenever we get time. Most of the day passes with a huge workload as wells as pressure for studying”. Again, a nurse shared her daily working plan as follows, “We have 3 types of working schedules, 8 hr shifts per week (5 days), 10 hr shifts per week (4 days) and 12 hr shifts per week (3 days). So, we work upto 36-40 hr in a week. But this whole time we have to continuously look after the patients. Sometimes, a single nurse like us has to look after 5 to 6 patients a day, but for surgical patients, we only look after one patient. If it is a very busy day with lots of patients, a single one of us looks after 8 to 9 patients at the same time. So, our workload becomes very intense during that time”. As all healthcare practitioners have to maintain a very long and busy schedule, it’s really hard to look after individual patients with great care.

#### Expectations about the digital system solution

Towards the end of each of the interviews with the participants, we directed the discussion towards our plan of designing and implementing a digital system solution. We tried to determine what functionalities the participants would like to be incorporated in a digital system for immobile and bedridden patients admitted in hospital cabins. During the discussion we also suggested some functionalities and requested them to share their opinions. We also tried to determine how a patient would like to interact with such a system. At first the discussion was mainly about the look and feel of the system. Since most of the participants did not have much idea about patient care systems, we shared with them the ideas about some of the systems based on existing literature and context of use. After that, a discussion about what type of system they would desire was initiated. For continuing the discussion, four different types of probable systems were proposed by us in the light of the literature review we had done earlier. These are: a) Embedded remote control based system, b) Mobile app based system, c) Computer Vision based system and d) Voice control based system.

##### a.Embedded remote control based system:

At first a probable embedded system solution was discussed, where different buttons would be present on the hardware for different functionalities. The entire apparatus would be like a remote. By pressing different buttons on the remote, the bedridden and immobile patient could perform different functions (for example turning off the light). This idea was liked by some of the patients. One patient said regarding this : “Having several functionalities inside a single remote would be awesome. It would help to decrease the dependence of the patients on external help.” However, this idea had some demerits which were pointed out by the healthcare practitioners. One of the doctors said about this matter: “A remote control for patient use is a good solution. But it requires one essential thing. The patients using it must be able to use their hands. It would not be of any use to the paralyzed and disabled patients.”

##### b.Mobile App based system:

When the idea of a mobile application based system was introduced, the participants had a mixed reaction about it. Some of the participants did not use smartphones. Also the problem of patients who are unable to use their hands remains an unresolved issue as well. Regarding the mobile app based system one of the intern doctors had the following opinion: “A mobile app for the patients is indeed a good idea. But the patients who are unable to use their hands will not be able to get any benefit from it. Also everyday several old patients are admitted in our hospital who do not know how to use smartphones.”

##### c.Computer Vision Based Interaction System:

For the discussion about the touch free computer vision based interaction system, a youtube video was shown to the patients at first, where hand gestures were being used to interact with such a system. Then their opinions were sought about. The touch free computer vision based system gave rise to a new interest in the minds of the participants. Since most of the participants were not familiar with it, they displayed more interest in this type of system. Some of the ex-patients wanted to see more videos of such systems. The doctors, intern doctors and nurses were skeptical but also equally interested about such systems. Regarding touch free systems one of the nurses had the following opinion: “Since the system will be touch free, there won’t be any hassle regarding placement and positioning of the system. This would also help to keep the system clean.” But again, the concern regarding the use of hands was brought to discussion by the participants. But when computer vision based, hands free, alternate means of interactions like using eye gaze, nose-tracking, etc were discussed, they began to show more acceptance towards such systems.

##### d.Voice control based system:

When the voice control based systems were discussed with the participants, they showed much interest. Regarding a voice control based system, one of the patients gave the following opinion: “I would be really delighted to be able to control my surroundings using my voice. It would be like the Iron Man movie” One of the patients had a concern regarding the voice based systems. Mentioning his concern, he said the following: “The idea of the system sounds amazing. But what if I am having a conversation with someone and the voice based system misinterprets my conversation as some commands? It can be a problem. Don’t you think so?” As a response to his concern, we described to him about smart home systems like Google Home and Alexa [49], where the user has to use a specific keyword before issuing any command. After this the patient seemed to have been convinced.

##### e.Functionalities desired from the system:

We asked all the participants about what type of functionalities they would like us to incorporate into the system. One of the functionalities which most of the participants wanted us to include was the ability to call someone in case of emergency. Regarding this one of the ex-patients provided the following opinion: “I would like to have an option which allows me to connect with a nurse or a family member. I would like to use that functionality in case of an emergency.” The healthcare practitioners also supported the inclusion of such functionality. One of the nurses had the following opinion: “It becomes really difficult at times to keep a close watch on everyone. Having an emergency call service would be really helpful for us to find the patients in need.” The doctors and intern doctors had similar opinions. The ex-patients also requested us to include a bed control function. Regarding this an ex-patient said: “Please keep the functionality to adjust the bed positions. As I have already mentioned, it really troubled me during my last stay in the hospital” The participants also wanted to include matters like light and temperature control. One of the nurses had the following opinion regarding this matter: “Many times we find patients calling us for trivial things like turning off the light or changing the temperature of the room. It would be great if the patients could do these tasks by themselves.”

##### f.Non-Functional Requirements:

Though none of the participants had much idea about non-functional requirements, during our discussion, some of their demands for non-functional requirements were reflected in their responses. One ex-patient said the following: “For the computer vision based system, I am a bit uncomfortable by the fact that I will be continuously in front of the camera. I am a bit worried about the privacy of the patients. Also are such systems secure? What if someone hacks into the camera and gets images from it?” Another concern about the system was about its responsiveness. One of the intern doctors who had some programming experience said in this regard, “If such a system is implemented. We would really expect it to be able to work fast as soon as a command is initiated. We do not want it to be laggy and slow.” Other than this, one of the doctors raised concern about the flexibility of the system. He said regarding this matter: “If a system is introduced in the hospital for patients, we would naturally want it to have more functionalities in the future. We would hope that the system would not be in a constant form and more functionalities can be introduced into it in the future.” In summary, the non-functional requirements of the system identified from the requirements elicitation study include privacy, security, real time responsiveness and flexibility.

#### Summary of the requirements elicitation study

Based on the interviews, we are able to draw some conclusions. Firstly, the intense pressure under which the healthcare practitioners continue to serve the patients have been greatly reflected during the interviews. Secondly, the problems faced by patients while being admitted in hospitals have been identified. The thematic analysis of the semi-structured interview revealed some of the major problems that are faced by admitted patients. Thirdly, the expectations of the patients, doctors, interns and nurses from a potential patient care system have been identified. Based on the findings from the requirements study, the design goals of the digital system solution were developed. And based on the design goals, an initial design of the system was generated. The system design is discussed in further details in the next subsection.

### The proposed system

The findings from the requirements elicitation process shows the necessity of a care-giving system for helping both the patients as well as the health care practitioners. Also while conducting the interviews, we obtained some ideas about the preferences and demands of the stakeholders from a potential care-giving system. In this subsection, at first the design goals for the care-giving system are established which are based on the findings from the requirements elicitation process. After that the components and workflow of the designed system are discussed.

#### Design goals

The design goals for the digital system solution has been set based on the findings from the requirements elicitation process. After much analysis of the findings from the interview, the following design goals have been set.

##### G1. Touch Free Computer Vision Based System:

During the interview, the participants mentioned different problems that can be faced by a touch based system (for example : remote controller, mobile application). Also the participants were more optimistic and enthusiastic when touch free computer vision based systems were discussed with them. Hence the design goal of the system is to make it fully computer vision based. This will address the concern related to the hygiene of the device. Furthermore, the system becomes independent of the patients’ position, distance and orientation to some extent.

##### G2. Supporting alternate means of interaction:

One of the main ways in which people interact with touch free systems is by using hand gestures. We initially wanted to base our system on hand gestures as well. But while conducting the interviews, the healthcare practitioners mentioned the cases about patients who are disabled and unable to use hands. Also voice based interaction was discussed with the participants. But one of the demerits of voice based interaction is that some patients may be unable to use it due to language barriers (not being adept in English Language or vocal impairment). Hence considering all the issues, we decided to make our system based on multimodal alternate means of interaction which includes: (i) hand gesture based interaction for the patients who are able to use their hands; (ii) nose-teeth based interaction for the disabled patients who are unable to use their hands and (iii) voice based interaction for patients who have no language barriers.

##### G3. A mobile application for the healthcare practitioners and family members:

The third design goal is the inclusion of a mobile application for the healthcare practitioners only. Using the mobile application, the healthcare practitioners can turn on or turn off the system. Also, the healthcare practitioners can set the interaction mode for the patient. Furthermore, in case of emergency, the patient can use the system to send an emergency alert message to the healthcare practitioner as well as to family members.

##### G4. Supporting frequently needed tasks:

From the interview, we managed to find out that the patients were facing difficulties in performing different kinds of tasks like turning on the fan, changing the temperature, adjusting the bed etc. Keeping these challenges in mind, the fourth design goal is to make the system capable of performing these frequently needed tasks. In the initial design of our system, we have kept the following tasks which the patient can perform by interacting with the system: turning on/off light, turning on/off fan, increasing/decreasing temperature, alerting nurse or family member for help and adjusting the bed by controlling its different motors.

#### Components of the system and workflow

Based on the requirements elicitation process and the design goals, the initial prototype of the system was designed. The initial prototype consists of mainly 3 components. These components are briefly discussed below.

##### Mobile Application for the healthcare practitioner:

**Fig. 1:**
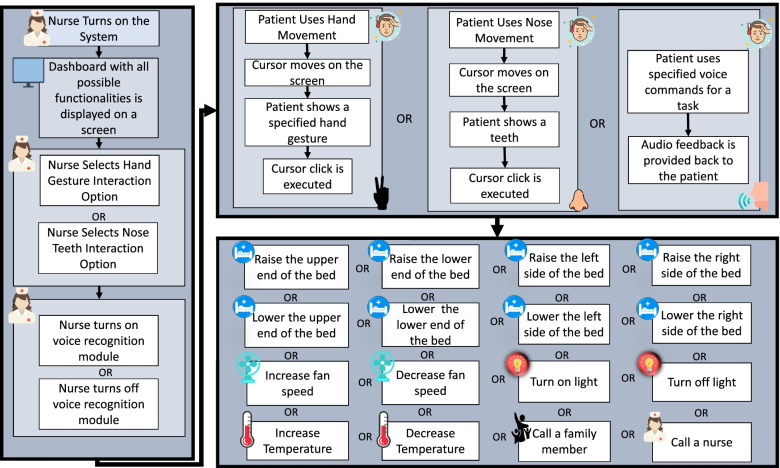
Workflow of the proposed system

An app for controlling the system will be installed in the smartphone of the healthcare practitioner in charge of the patient. Using the mobile application, the system can be turned on or turned off. Also the mobile app provides the option to select interaction mode for the patient. Furthermore, using the mobile app, the healthcare practitioner can turn on or turn off voice interaction for the patient as well. In Fig. 1, the mobile application component has been shown as a part of the workflow diagram.

##### Interaction Module

This component of the system is dedicated towards understanding different modes interaction of the patients. The interaction module consists of the three sub modules. These are described below:

###### a.Hand gesture based interaction sub-module

This sub module is responsible for detecting and analyzing hand gestures from the patient. Using a camera, images of the patient will be taken continuously. A cursor with some options will be displayed on a screen. Using predefined hand gestures and hand movement, the patient will try to select an option by clicking on it. This module will be responsible for detecting the patient’s hands and for understanding what gesture is shown by the patient. If a gesture for clicking is shown, this module will interpret it as a clicking gesture and hence the clicking will be performed on the selected option.

###### b.Nose Teeth based interaction sub-module

This sub module is responsible for detecting the nose movements of the patient. Using a camera, images of the patient will be taken continuously. A cursor with some options will be displayed on a screen. Using nose movement for cursor movement and by showing teeth for executing a click, the patient will try to execute an option.

###### c.Voice based interaction sub-module

This sub module is responsible for detecting voice commands from the patient. A pre-specified name will be set for this module, for example “John”. When the patient wants to use the voice based sub module, the patient has to talk to it by calling its preset name. For example “John turned on the fan”. The voice module will convert the patient’s speech contents to text contents. After that, by analyzing the contents of the converted text, the voice module will issue commands to the task execution module which is discussed in the next subsection.

##### Task executing module

The task executing module will be responsible for executing the tasks that were set in the design goal G4. The interaction module will send the selected option to this module for execution. Necessary hardware implementation will be done so that this module can control light, fan, thermostat and the motors of the patient’s bed. Also this module is able to send alert messages to the healthcare practitioner of a family member of the patient. In Fig. 1, the workflow of the design is shown with all the major components in it.

### Development of the system

For the development of the hand gesture based interaction subsystem, two types of functions were needed. At first a function was needed for identifying the position of the patient’s hand in the image. Next, a function for identifying the gesture of the hand in the image was needed. For the development of the nose teeth based interaction subsystem, three types of functions were needed. At first a function was needed for identifying the position of the patient’s face in the image. Next, a function for identifying the position of the patient’s nose in the image was needed. Lastly, a function for determining whether the patient is showing teeth or not was needed. For the development of the voice based system, we required a function which would take human speech as input and provide the textual representation of the speech. From the generated text, certain keywords could be searched to determine the meaning of the speech.

While implementing the hand gesture based interaction system, for identifying a patient’s hand position in the image, the CNN based YOLO algorithm has been used [39, 50–52]. When speed is the main priority, this algorithm performs better for real time detection and tracking compared to the other CNN based detection and tracking algorithms [53, 54]. For classifying the hand gesture shown by the patient, we have used a CNN based classifier with softmax regression function because CNN has strong ability for feature extraction, accuracy and adaptability [55]. For building this CNN using transfer learning [56–58], the architecture of Nasnet Mobile [38, 59] was used to reduce training time.

Similarly, for the nose-teeth based interaction system, in order to identify the position of the patient’s face and nose, the YOLO algorithm has been used again. For determining whether a patient is showing teeth or not, CNN based classifier with softmax regression function was again used for the same reasons stated above. For building this CNN, transfer learning was used again to utilize the Nasnet Mobile architecture.

For the voice-based interaction system, for converting the patient’s speech into corresponding text, Google Voice API [60] was used. From the generated text, specific keywords are to be searched to determine what the patient is wanting to do.

Thus, for the development of the system, a total of six machine learning models were needed to be developed. These models are *YoloNetForHand*, *YoloNetForFace*, *YoloNetForNose*, *HandClassifier*, *TeethClassifier* and *VoiceToTextConverter* for hand detection and tracking, face detection and tracking, nose detection and tracking, hand gesture detection, teeth detection and speech to text conversion respectively.

For the model *VoiceToTextConverter*, google voice API was used. The rest of the models are implemented by collecting data, preparing dataset and training the models. In the following sub-subsections, the collection and preprocessing of the dataset is described.

#### Image data acquisition and preprocessing

For training *YoloNetForFace*, at first the images of 10,000 human faces were collected from the Open Images V6 database [61]. Each image had corresponding co-ordinates specifying the position of the human face in the image. Using the coordinates, the position of face in the images can be labelled. Similarly, from the same source [61], for training *YoloNetForNose*, images of 10,000 human noses along with corresponding coordinates for the nose positions were collected. For creating the teeth classifying convolutional neural network *TeethClassifier*, the 5,000 images of the mouth (teeth shown and teeth not shown) were used for preparing the dataset.

For training *YoloNetForHand* at first the images of 10,000 human hands were collected from the website Open Images V6 [61]. Each image had corresponding co-ordinates specifying the position of the human hand in the image. Using the coordinates, the position of hand in the images can be labelled. For creating the hand gesture classifying convolutional neural network *HandClassifier*, the 5,000 images of the hand(hand closed and hand opened) were used for preparing the dataset. The summary of the collected data is displayed in Table 4.

**Table 4:** Summary of collected data

Model Name	Summary of collected data	Frequency	Source
*YoloNetForHand,*	Images with human hands with corresponding hand coordinates	10,000	Open Images V6 [Ref]
*YoloNetForFace,*	Images with human faces with corresponding face coordinates	10,000	Open Images V6 [Ref]
*YoloNetForNose,*	Images with human nose with corresponding nose coordinates	10,000	Open Images V6 [Ref]
*HandClassifier,*	Images of Open hands and closed hands	5,000	Collected in real time using *YoloNetForHand*
*TeethClassifier*	Images of mouth region (showing teeth and not showing teeth)	5,000	Collected in real time using YoloNetForFace

#### Developing the interaction systems

Using the datasets prepared that have been mentioned in the previous sub-subsection, the five mentioned neural networks *YoloNetForHand, YoloNetForFace, YoloNetForNose, HandClassifier* and *TeethClassifier* were developed.

**Fig. 2:**
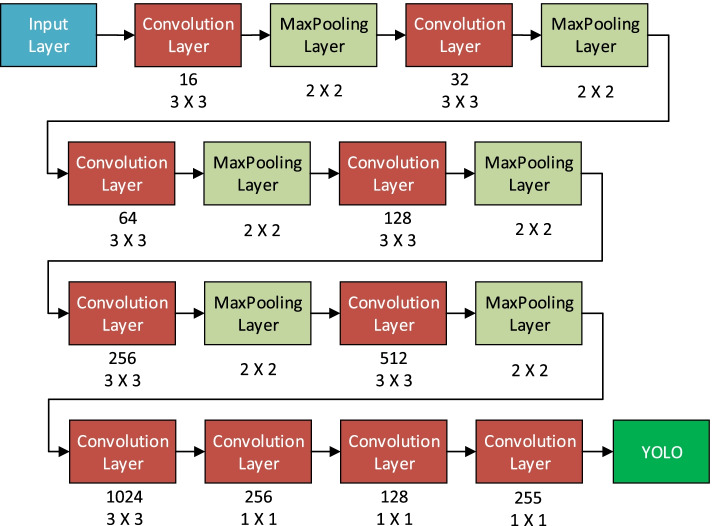
YOLO based CNN architecture

For developing the YOLO based CNNs (*YoloNetForHand, YoloNetForFace, YoloNetForNose, HandClassifier*), the architecture of the YOLO version 3 tiny model [62] was used because it requires less storage space and works fast while processing data in real time. The architectural design of the used YOLO based CNN is shown in Fig. 2.

As discussed in the Methodology section, the YOLO architecture, consists of one Input Layer, ten Convolutional layers and six Pooling Layers performing Maxpooling operations [63]. The feature map obtained as output from the final convolutional layer is used as input for the YOLO algorithm. Upon the execution of the YOLO algorithm, the type of the detected objects and the position of the detected objects are obtained as outputs.

**Fig. 3:**
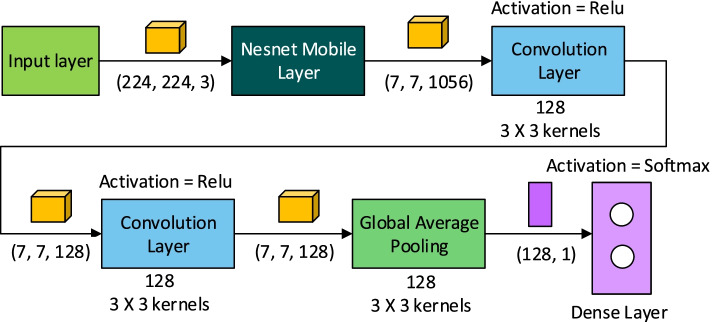
CNN Classifier Atchitecture

For developing the CNN classifiers, transfer learning [56, 57] has been used and the hence the architecture and weights of the model Nasnet Mobile [38, 38] has been utilized. In Fig. 3, the architectural design adopted for the CNN classifiers has been shown.

Except the final output Dense Layer of the Nasnet Mobile model, all the other layers have been subjected to transfer learning. These layers of the Nasnet Mobile model were not subjected to training for preserving its feature detection capabilites. The feature maps obtained as output from the Nasnet Mobile layers were provided to two consecutive convolutional layers. The weights of these convolutional layers were trained in order to adapt the model for the dataset of this research. Finally a Global Average Pooling layer has been used to reduce the dimention of the 3 Dimensional feature map from the final convolutional layer into 1 Dimensional feature map. This 1 Dimensional feature map can be used by a Dense layer for classification and hence the final layer of the CNN classifiers is a Dense layer with softmax activation function.

In the following sub-subsection, the development of the interaction systems including the training of the models and integration into the system are discussed.

#### Development of the nose teeth based interaction system

**Fig. 4:**
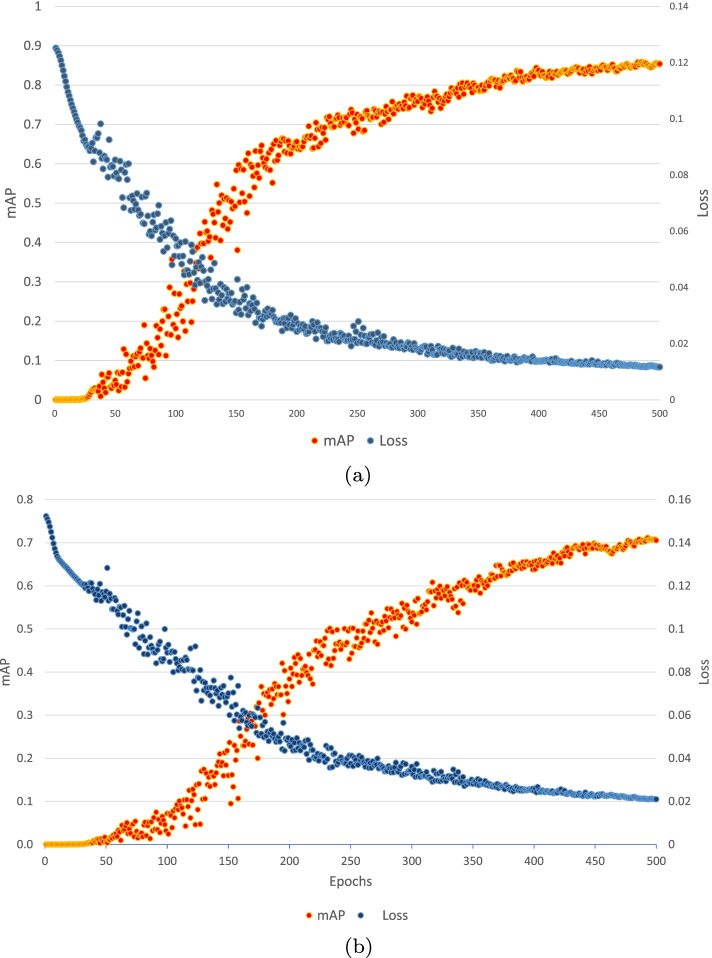
**a** Face YOLO model graph, **b** Nose YOLO model graph

The nose-teeth based interaction system consists of the three neural network models which are *YoloNetForFace*, *YoloNetForNose* and *TeethClassifier*. Both of the YOLO based models have been trained for 500 epochs [64, 65]. For training both of these models, the batch size was set to 64, the learning rate was chosen to be 0.0005 and the loss function used was mean squared error. The final accuracy metric value mAP for the two models were found as 0.8541 and 0.70531 respectively [66]. Figure 4a and 4b displays plotting of loss and mAP for the models *YoloNetForFace* and *YoloNetForNose* respectively. The third neural network *TeethClassifier* is a convolutional neural network for classifying images into two classes. One class consists of images where the mouth is opened and teeth are shown and the other class consists of images where the mouth is closed and teeth are hidden. This model was trained for 100 epochs with a batch size of 32 and learning rate of 0.0001.The training of this model resulted in a binary cross entropy loss [67] value of 0.02 and an accuracy of 0.994141.

**Fig. 5:**
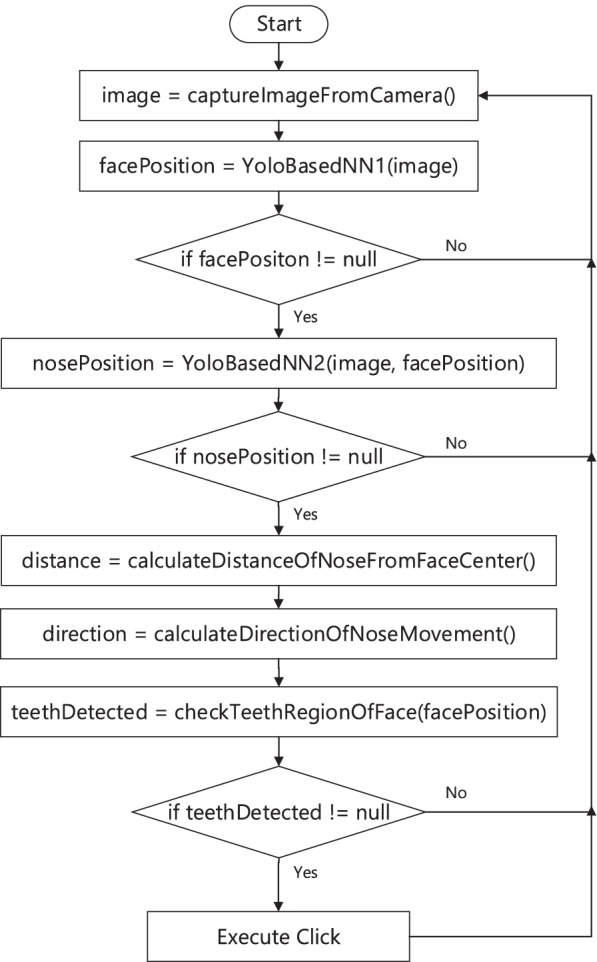
Workflow of nose-teeth based interaction system


The flowchart in Fig. 5 and the pseudocode in Algorithm 1 describes how these models have been used for implementing the teeth based interaction sub system. At first a camera is used to capture images of the patient in real time. Then using *YoloNetForFace*, the position of the face of the patient is identified in the image. After that using *YoloNetForNose*, the position of the nose of the patient is identified in the image. Then based on the relative distance between the center of the face and the tip of the nose, the displacement and direction values are calculated. The displacement variable determines at what speed the cursor is to be moved and the direction variable determines towards which direction the cursor is to be moved. Finally the *TeethClassifier* is used to detect whether the patient is showing his/her teeth or not. If teeth is detected by the *TeethClassifier*, then a cursor click is executed on the screen and the option on which the cursor is located is selected for execution.

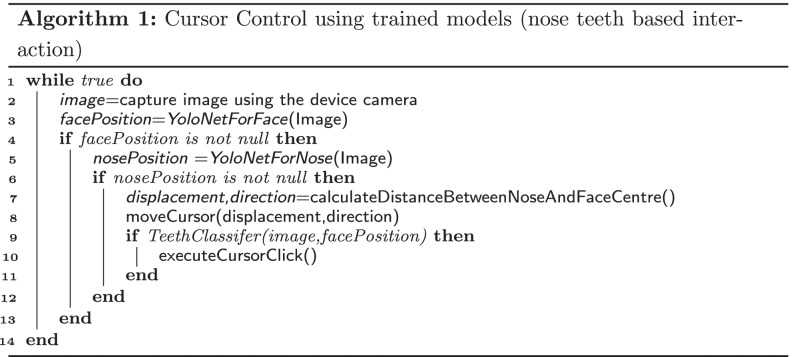


#### Development of the hand gesture based interaction system

**Fig. 6:**
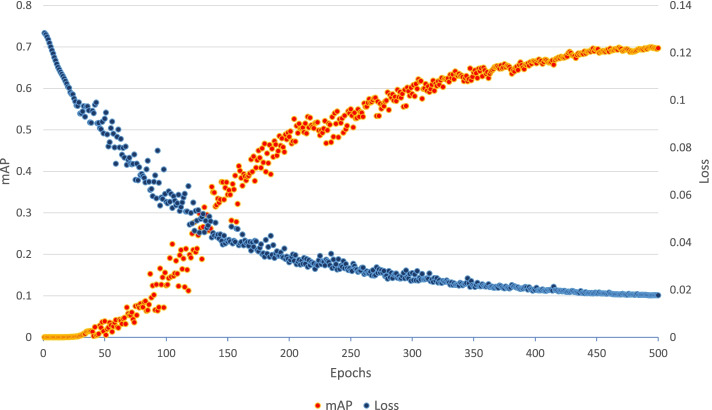
Hand YOLO model graph

The hand gesture based interaction system consists of the 2 neural network models which are *YoloNetForHand* and *HandClassifier*. The *YoloNetForHand* model has been trained for about 500 epochs [64]. The batch size for this model was set to 64, the learning rate was chosen as 0.0005 and the loss function selected was mean squared error. The final value of the metric mAP for this model was found to be 0.69713 [66]. Figure 6 displays plotting of loss and mAP for the model. The second neural network *HandClassifier* is a convolutional neural network for classifying images into two classes. One class consists of images where the hand is opened and the other class consists of images where the hand is closed. This model was trained for 100 epochs with a batch size of 32 and learning rate of 0.0001. The training of this model resulted in a binary cross entropy loss value of 0.07 and an accuracy of 0.993036.

**Fig. 7:**
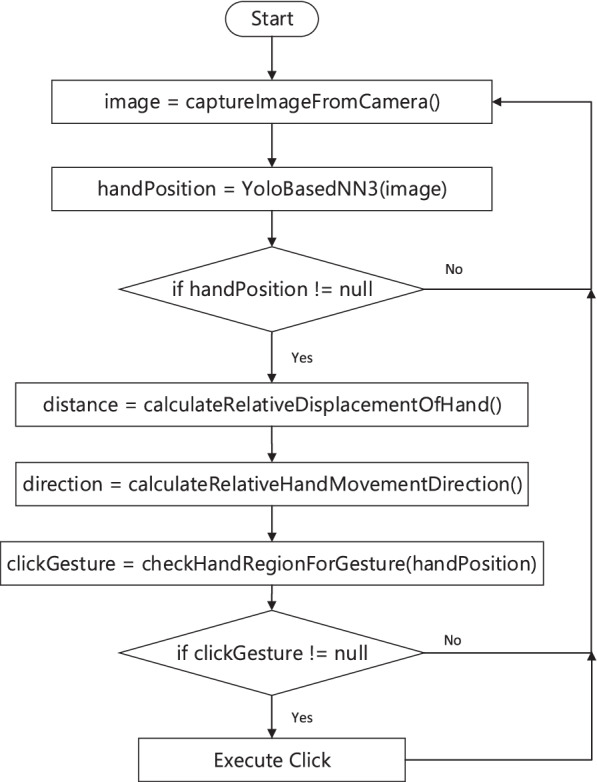
Workflow of Hand based interaction system


The flowchart in Fig. 7 and the pseudocode in Algorithm 2 describes how the two models have been used for implementing the teeth based interaction sub system. At first a camera is used to capture images of the patient’s hand in real time. Then using *YoloNetForHand*, the position of the hand of the patient is identified in the image. Then based on the relative movement of the hand in a direction, the displacement and direction values are calculated. The displacement variable determines at what speed the cursor is to be moved and the direction variable determines towards which direction the cursor is to be moved. Finally the *HandClassifier* is used to detect whether the patient is showing open hand gesture or closed hand gesture. If closed hand gesture is detected by the *HandClassifier*, then a cursor click is executed on the screen and the option on which the cursor is located is selected for execution.

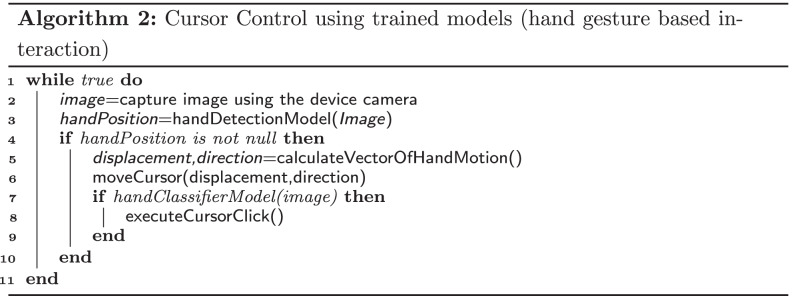


#### Statistical analysis of the deep learning models

To evaluate the developed deep learning models for their effectiveness and robustness, statistical analysis was conducted. The classification CNNs and the YOLO based CNNs were analyzed using different sets of metrics that have been found to be widely used in existing deep learning based researches.

**Statistical Analysis of the Classification CNNs:** For evaluating the classification CNNs i.e. the *HandClassifier* and the *TeethClassifier*, the metrics used for the statistical analysis were accuracy, precision, recall, f1 score, cohen’s kappa and Area Under the Curve of Reciever Characteristic Operator (ROC-AUC) [68, 69].

These metrics are positive metrics and hence, the more the value of these metrics, the better will be the performance of the model being evaluated. For the evaluation of the classification CNNs, 1200 hand gesture images and 1200 mouth images were prepared using the webcam and labelled. The images were subjected to preprocessing operations including rescaling the pixel values, resizing the image resolution and grayscaling. Afterwards, the images were used as inputs for the two CNN classifiers and the prediction values were stored in a file. Then based on the prediction values and the label values, the statistical analysis was conducted using the six aforestated metrics. Furthermore, the images used for training were also preprocessed using the same process and statistically analyzed using the same metrics for comparison purposes.

In the Table 5, the results of the statistical analysis has been presented for both the training and the testing datasets.

**Table 5:** Statistical Analysis of CNN classifiers

Metrics	TeethClassifier		HandClassifier	
Models	Training	Testing	Training	Testing
Accuracy	0.993036	0.940188	0.994141	0.896552
Precision	0.997508	0.973428	0.998035	0.972441
Recall	0.988477	0.904527	0.990253	0.815182
F1 Score	0.992972	0.937713	0.994129	0.886894
Cohen’s Kappa	0.986070	0.880336	0.988281	0.792934
ROC-AUC	0.993015	0.940028	0.994148	0.896153

The analysis of the statistical data shows that both of the models performed better on the training dataset compared to the testing dataset. The model *TeethClassifier* did not overfit as there is no significant variation of the metric values when the training and testing results are compared. Only the metric value of Cohen’s Kappa varies to some extent (by about 0.1). However, the model *HandCLassifier* seems to have overfitted a bit because other than the precision values, there is significant variation of performance based on the training dataset and the testing dataset metrics.

The statistical analysis further shows that on the training dataset, both the *TeethClassifier* model and the *HandClassifer* model performs almost equally well. However, on the testing dataset, the model *TeethcClassifier* performed considerably better than the model *HandClassifier* which can be attributed again to the fact that the *HandClassifier* model might have overfitted.

**Statistical Analysis of the YOLO based CNNs:** For evaluating the YOLO based CNNs i.e. the *YOLONetForHand*, *YoloNetForFace* and *YoloNetForNose* the metrics used for the statistical analysis are, precision, recall [68, 69], mean average precision (mAP) [66, 70], box loss and objectness loss [71].

Among the stated metrics, mAP, precision and recall are positive metrics and thus, the more the value of these metrics, the better will be the performance of the model being evaluated. On the other hand, box loss and objectness loss are negative metrics i.e. the less the value of these metrics, the better will be the performance of the model being evaluated.

For the evaluation of the YOLO based CNNs, 1200 human hand images, 1200 human face images and 1200 human nose images were collected along with the output values from the online open source database [61] used for training the models. The images were subjected to preprocessing operations including rescaling the pixel values, resizing the image resolution and grayscaling. Afterwards, the images were used as inputs for the respective YOLO based CNNs and the prediction values were stored in a file. The predicted values included the type of the detected objects and the location of the detected object in the image. Then based on the prediction values and the label values, the statistical analysis was conducted using the five aforestated metrics. Furthermore, the images used for training were also preprocessed using the same process and statistically analyzed using the same metrics for comparison purposes.

In the Table 6, the results of the statistical analysis has been presented for both the train and the test datasets.

**Table 6:** Statistical Analysis of YOLO based CNNs

Metrics	YoloNetForHand		YoloNetForFace		YoloNetForNose	
Models	Training	Testing	Training	Testing	Training	Testing
mAP	0.69713	0.67632	0.8541	0.79121	0.70531	0.65832
Box Loss	0.015149	0.020341	0.009688	0.021681	0.010975	0.022861
Objectness Loss	0.018167	0.023082	0.014921	0.029593	0.031975	0.031567
Precision	0.98613	0.931751	0.98314	0.955214	0.97992	0.961431
Recall	0.99371	0.98651	0.99611	0.966221	0.98543	0.95443

The statistical analysis shows that all the YOLO based CNNs performed better on the training dataset compared to the testing dataset. Nevertheless, the models did not overfit as the metrics values obtained for the testing datasets are not much deviated from that of the training datasets. On both the training dataset and the testing dataset, the model *YoloNetForFace* performed better in terms of mAP. For the metric box loss as well as objectness loss, *YoloNetForFace* performed better on the training dataset and *YoloNetForHand* performed better on the testing dataset. The highest value of precision was obtained by the model *YoloNetForHand* on the trainig dataset and the model *YoloNetForNose* obtained the highest precision value on the testing dataset. In case of the metric Recall, the highest value was obtained by the model *YoloNetForFace* on the training dataset and the model *YoloNetForHand* on the testing dataset.

#### Development of the voice based interaction system

For the voice based interaction system, Google Voice Api was used. No training of models was required in this case. For this system, at first human voice is taken as input. Then using the api, the voice is converted into text. Then using regular expression [72] keywords are extracted from the text. Based on the extracted keywords, one of the tasks displayed in the Graphical User Interface are selected for executing. The pseudocode for the voice based interaction system is provided below in Algorithm 3.


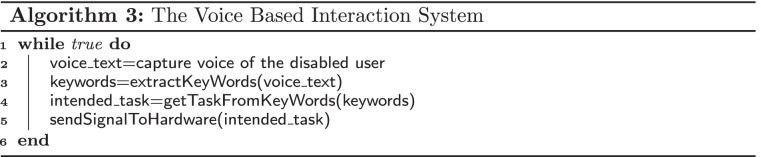


#### The user interface and prototype bed

**Fig. 8:**
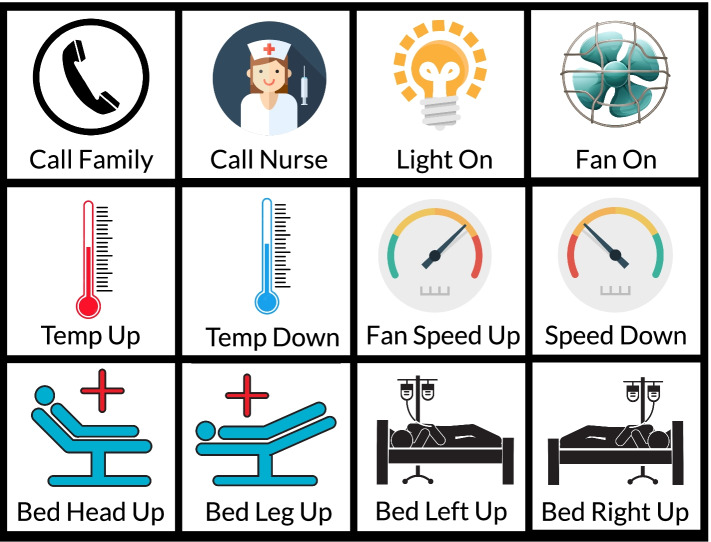
GUI for the developed system

A Graphical User Interface (GUI) shown in Fig. 8 was developed using Python Tkinter [73], integrating all the three models discussed above. Also the google’s voice API was added so that patients can avail all the three interaction systems. An LCD monitor will be placed in front of the patient. The GUI will be displayed on that monitor. The GUI is interactive and user friendly. When an action is being triggered through any of the aforementioned interactions, the corresponding component flashed in the GUI responds accordingly. Users also get to know which functions are currently active and which are not. In addition to that, an android application was also developed, which is to be installed in the phone of the concerned nurse or family members. The app was built using Firebase Real-time Database [74, 75]. The patient can alert the nurse or family members by clicking on the call options in the GUI or by using voice command.

**Fig. 9:**
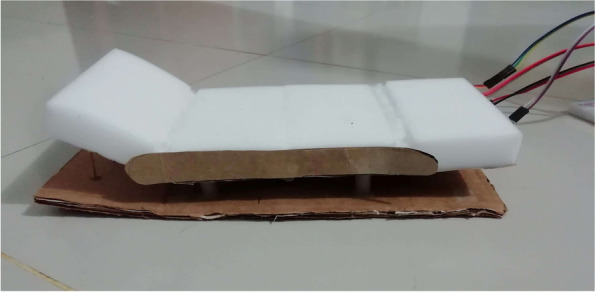
Developed prototype bed

**Fig. 10:**
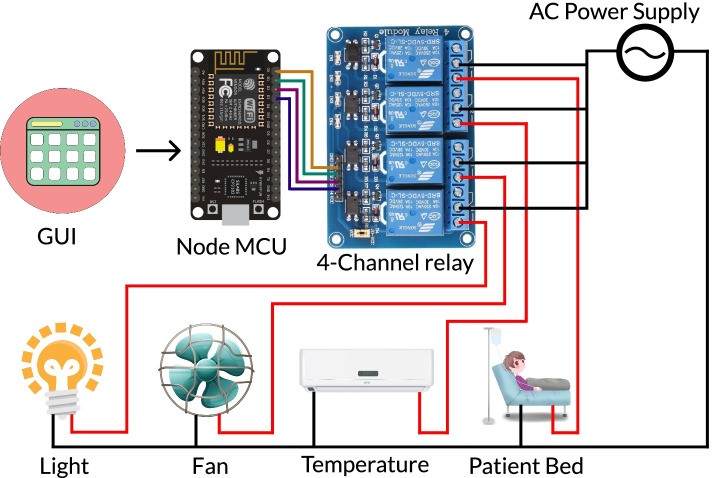
Circuit diagram for hardware connections

The prototype of the developed system included hardware components dedicated to controlling the surrounding environment e.g. light, fan, room temperature, patient bed etc. For this initial version of the system, a prototype of the patient bed was implemented as shown in Fig. 9. To establish wireless connection between the patient computer and the hardware devices, a NodeMCU (ESP8266) [76] chip was used. Patient computer is connected with the NodeMCU through Wifi. A 4-channel relay was used to control the AC devices. This relay is connected with the NodeMCU. Control commands to control the surrounding environment including light, fan, room temperature and patient bed etc. are delivered from GUI to the NodeMCU through Wifi. A Diagram depicting the circuit of the hardware connection is shown in Fig. 10.

### Results of the system evaluation

The system evaluation procedure described in the “Methodology” section yielded qualitative and quantitative data as results. Based on the generated data, the efficiency, effectiveness and usability of the system has been measured.

For ease of representation, the hand based interaction, nose-teeth based interaction and voice based interaction are represented using the short forms HB, NTB and VB respectively in Table 7, where the measure of the effectiveness of the system is showed.

**Table 7:** Measure of effectiveness of the developed system

Task	Successful Execution			No of attempts (mean,std)		
	HB(n=3)	NTB(n=5)	VB(n=4)	HB	NTB	VB
T1	100%	100%	100%	1.66, 1.15	2.01, 1.22	1.66, 1.15
T2	100%	100%	100%	1.70, 1.20	2.23, 1.34	1.57, 1.29
T3	100%	100%	100%	1.61, 1.11	2.31, 1.11	1.61, 1.31

The effectiveness of the system was tested by checking whether the patients were able to execute the tasks successfully or not. For each task category, the average number of attempts and the standard deviation of attempts were recorded. The patients were able to execute the tasks successfully. However, the number of attempts taken to execute the tasks varied. The average number of attempts were higher for the nose teeth based interaction. But the standard deviation is also high for the nose teeth based interaction and hence the effectiveness of this interaction varies vastly between participants who took part.

The efficiency of different interaction methods was determined, by observing the task completion time for different task categories using different interaction methods and by keeping track of the number of times a patient asked for help. (see Table 8). It was found that the mean value was highest for nose teeth based interaction. During the conduction of the experiment none of the participants asked for any help as they were provided with explanations and demonstrations before starting the experiment.

**Table 8:** Efficiency

Task	Task completion time(mean,std)		
	HB	NTB	VB
T1	(6.725,5.349)	(12.38,5.56)	(7.23,1.61)
T2	(6.812,5.143)	(11.725,2.349)	(10.725,5.349)
T3	(7.131,2.112)	(10.711,5.349)	(11.725,5.349)

After the completion of the testing, feedback was obtained from all the patients. A semi-structured interview was conducted with each of the 12 participants. The interview sessions were recorded after taking their permission. After that, a thematic analysis was performed [36, 37] on the collected recordings. The outcome and findings from the thematic analysis is presented below:

#### Requirement of training

It was identified from the interview that the participants felt that they would require some training before being accustomed to using the system. They also mentioned some tutorial videos for explanation. Regarding this matter, one of the patients said “The use of your system is straightforward, but still I think a tutorial or training video would be great for it. Also since when we are really sick, we face difficulty in understanding stuffs. So some more time should be given for teaching us how to use it.”

#### Supporting the patient’s language

The voice based interaction, as discussed earlier, was built using the google voice API, where a participant’s speech is converted into corresponding English text. This interaction method is usable only if the patient is able to speak using the English language. But patients who are unable to speak English won’t be able to make use of it. Regarding this, one of the participant patients said the following “I opted to use the nose-tracking based interaction, but I think I would personally prefer the voice based one. But I don’t know how to speak in English, so it would not be of use to me.”

#### Useful for supporting disabled people

The developed system was praised by the patient as it would be usable by patients who are physically disabled, for example people without hands. Also it is usable by patients who are unable to speak. Regarding this one of the patients with broken arms said “This system would be really helpful for me. Being unable to use my hands, I am facing limitations”. Similarly another patient praised the system saying the following “It is really thoughtful of you all to think about those people who are unable to use their hands or are paralyzed.”

#### Reduces the need for external help

The developed system was further praised for its capacity to help patients be more independent from external help. It makes an admitted patient able to control his/her surroundings to some extent. It also is capable of adjusting the patient’s bed. Regarding its usefulness, one of the patients had the following opinion: “With this, I will be able to do many tasks on my own while staying in my bed. It makes me more independent to some extent.”

Reduces the pressure on healthcare providers: One of the patients pointed out the fact that, the developed prototype once implemented fully will be able to reduce the prevalent load and pressure on the healthcare sector. He said regarding this “I was admitted in a Corona ward before and I have seen how the healthcare workers work hard to take care of us during dire times. Your system will be a great tool for them.”

## Discussion

In this research, the requirements of patients and healthcare practitioners in the context of a hospital cabin has been elicited and analyzed. It is seen that there are no prior studies that explicitly focus on identifying the requirements of a hospital admitted, bedridden and immobile patient. Most of the admitted patient care systems were developed for monitoring purposes. [9, 16, 21, 22]. Furthermore, though different systems have been developed to allow bedridden and disabled patients interact using alternate means of interaction [10, 11, 27, 28, 30, 32], none of the research works were found to focus on incorporating multimodal means of interaction. Also, none of the earlier studies conducted requirements elicitation from the hospital admitted, bedridden and immobile patients at field level and the their identified requirements where based on assumptions to a large extent. In this work, we have developed a multimodal interaction system based on hand gesture, nose-teeth and voice commands using deep learning. Evaluation of the system has been done for verifying its efficiency, effectiveness and usability.

Henceforth, this research has several implications. Firstly, the interview dataset obtained during the requirements elicitation study contains inputs from all the stakeholders directly related to the hospital cabins or wards. Hence it can serve future research works and innovations for hospital admitted patients. Secondly, the system developed as a result of this research can aid sick or disabled people not only in the hospital cabins or wards but also at households, nursing homes, rehabilitation centres, etc. It can help to provide cost-effective assistance and reduce the prevailing pressure in the healthcare sector. Finally, the evaluation results obtained from the evaluation study can serve as a benchmark and thus, help researchers for comparison purposes in similar future works.

The research has a few limitations as well. Firstly, for using the voice based interaction, a patient is required to speak in English Language. Secondly, we did not consider the impact of using the camera continuously on power usage. Thirdly, though the YOLO algorithm was selected for this research via theoretical comparisons, no statistical analysis was performed to compare YOLO with the other state of the art algorithms based on cross-validation procedure, performance measures and significance non-parametric tests. Finally, a real hospital bed was not used during testing and evaluation, a prototype bed representing the functionalities of a real bed was used.

## Conclusion

The aim of this study was to help hospital admitted, bedridden and immobile patients. Requirements elicitation study was conducted to reveal the requirements of these patients which will serve as a rich source of information for upcoming related researches. Computer vision and deep learning technologies were used to address these requirements and implement a multi-modal interaction system for hospital cabins for such patients. Finally an evaluation system was conducted to determine the efficiency, effectiveness and usability of the developed system.

Compared to the previous researches, this study has several advantages. Unlike most of the previous works for the hospital admitted patients [9–11, 16, 21–23], this research is based on data that has been collected through requirements elicitation study at the field level. Moreover, several of the previous works [9, 16, 21, 22] used different sensors that required to be attached with the patients’ body but in this research no such invasive measures are required and only image data in real time is needed. Again, unlike most of the previous researches which supported a single mode of interaction [11, 27, 28, 30, 32, 33], this research focused on multimodal interaction to support patients at different levels of the disability spectrum. Furthermore, most of the previous works [27, 28, 30, 32, 33] did not adopt deep learning technologies unlike the proposed system.

However, this system has some disadvantages as well. The doctors and nurses do not have much control over the system and need to go to the patient’s cabin physically for any kind of necessary intervention. Unlike the BCI based systems [10, 23], the system is not applicable for patients who have become completely disabled. Furthermore, unlike the previous researches [16, 21], the proposed system is not capable of extracting information like temperature, heartbeat or other medical data.

This research has introduced some scientific novelties as well. Firstly, as per the authors’ knowledge no other multi-modal interaction system has been developed based completely on deep learning technologies which is further supported by the literature review. Secondly, no other researches were found to systematically analyse the requirements and challenges of a hospital patient cabin through empirical studies. Finally, no other system has been found during the literature survey where three or more interaction systems have been brought into one common platform. We believe that this system will make things easier for hospital admitted patients and the dedicated healthcare practitioners.

In the future works, the proposed system will be evaluated by integrating real hospital beds. We will also evaluate the proposed system so that the use of power consumption can be comprehended. Furthermore, we intend to perform a full statistical analysis of the YOLO algorithm by comparing with other state-of-art algorithms based on cross-validation procedure, performance measures and significance non-parametric tests.

## Data Availability

The datasets generated during and analyzed during the current study are not publicly available due to the fact that we are still using it for another publication, but are available from the corresponding author on reasonable request.

## Abbreviations

CNN: Convolutional Neural Network
YOLO: You Only Look Once
mAP: mean Average Precision
